# Promoting mental health and wellbeing in schools: examining Mindfulness, Relaxation and Strategies for Safety and Wellbeing in English primary and secondary schools: study protocol for a multi-school, cluster randomised controlled trial (INSPIRE)

**DOI:** 10.1186/s13063-019-3762-0

**Published:** 2019-11-21

**Authors:** Daniel Hayes, Anna Moore, Emily Stapley, Neil Humphrey, Rosie Mansfield, Joao Santos, Emma Ashworth, Praveetha Patalay, Eva-Maria Bonin, Bettina Moltrecht, Jan R. Boehnke, Jessica Deighton

**Affiliations:** 1 Evidence Based Practice Unit (EBPU), University College London and Anna Freud National Centre for Children and Families (AFNCCF), The Kantor Centre of Excellence, 4-8 Rodney Street, London, N1 9JH UK; 2 Manchester Institute of Education, The University of Manchester, Manchester, UK; 3 MRC Unit for Lifelong Health and Ageing and the Centre for Longitudinal Studies, University College London, London, UK; 4 Care Policy and Evaluation Centre (CPEC), London School of Economics and Political Science, London, UK; 5 School of Nursing and Health Sciences (SNHS) and the Dundee Centre for Health and Related Research (DCHARR), University of Dundee, Dundee, UK

**Keywords:** Adolescent, Young person, Children, Cluster randomised controlled trial, Mental health, Wellbeing, School-based

## Abstract

**Background:**

There are increasing rates of internalising difficulties, particularly anxiety and depression, being reported in children and young people in England. School-based, universal prevention programmes are thought to be one way of helping tackle such difficulties. This protocol describes a four-arm cluster randomised controlled trial, investigating the effectiveness of three different interventions when compared to usual provision, in English primary and secondary pupils. The primary outcome for Mindfulness and Relaxation interventions is a measure of internalising difficulties, while Strategies for Safety and Wellbeing will be examined in relation to intended help-seeking. In addition to the effectiveness analysis, a process and implementation evaluation and a cost-effectiveness evaluation will be undertaken.

**Methods and analysis:**

Overall, 160 primary schools and 64 secondary schools will be recruited across England. This corresponds to 17,600 participants. Measures will be collected online at baseline, 3–6 months later, and 9–12 months after the commencement of the intervention. An economic evaluation will assess the cost-effectiveness of the interventions. Moreover, a process and implementation evaluation (including a qualitative research component) will explore several aspects of implementation (fidelity, quality, dosage, reach, participant responsiveness, adaptations), social validity (acceptability, appropriateness and feasibility), and their moderating effects on the outcomes of interest, and perceived impact.

**Discussion:**

This trial aims to address important questions about whether schools’ practices around the promotion of mental wellbeing and the prevention of mental health problems can: (1) be formalised into feasible and effective models of school-based support and (2) whether these practices and their effects can be sustained over time. Given the focus of these interventions on mirroring popular practice in schools and on prioritising approaches that present low-burden, high-acceptability to schools, if proved effective, and cost-effective, the findings will indicate models that are not only empirically tested but also offer high potential for widespread use and, therefore, potentially widespread benefits beyond the life of the trial.

**Trial registration:**

ISRCTN16386254. Registered on 30 August 2018.

## Background

Well-established estimates in the United Kingdom suggest that one in eight children and young people experience mental health problems [1] and that these may be with associated with costly long-term consequences [2–4]. In the absence of effective or widespread processes for identifying those who experience mental health problems, or those likely to be at risk of such difficulties in the future, there has been an increasing focus on universal approaches to supporting children’s mental health and wellbeing. These universal interventions can act as a means to prevent the emergence of mental health problems and to intervene early in the emergence of any difficulties [5, 6]. Schools are often viewed as a universal point of access to children and young people, offering an important opportunity to embed prevention and early intervention programmes [7, 8]. A number of reviews point to the effectiveness of school-based mental health programmes for the prevention and early intervention [9], especially for depression [10], anxiety [11] and behaviour problems [12]. While existing evidence makes a good case for the effectiveness of universal school-based interventions [11, 13], a number of areas require further clarity.

Firstly, with the exception of a small number of UK-based programmes [14, 15], the basis for current practice in the UK is often research evidence originating from other countries, predominantly the US, with social and emotional learning (SEL) programmes such as Incredible Years [16] and PATHS [17] being highly popular. Other than the Incredible Years programme, which has been rigorously tested in a UK setting [18], rigorous and consistent evidence for SEL programmes’ effectiveness is sparse. Additionally, there are indications that some programmes do not always translate well when implemented beyond their countries of origin [19, 20].

Secondly, a scoping review of existing practice indicates a heterogeneous range of mental health support offered in schools, much of which is either novel, not based on tried and tested programmes, or involves a high level of adaptation from existing evidence-based approaches [21, 22]. However, reasons for adaptation are often logical; these programmes, which have not been designed for the UK school context, frequently require tailoring for suitability and feasibility, which may be beneficial to outcomes. However, such adaptation also carries a risk of significantly ‘watered-down’ implementation, which limits impact [23].

Three interventions were selected by the Department for Education in England to be developed for the current trial. The basis for selection was that these either: (a) were popular approaches being adopted by schools and, therefore, likely to have high acceptability and feasibility, as well as potential for wider adoption if found to be effective; or (b) showed early promise but currently lacked a robust evidence base, specifically regarding implementation in schools. The interventions were: (1) Mindfulness Practices, (2) Relaxation and (3) Strategies for Safety and Wellbeing, based on the principles of ‘Protective Behaviours’(PB). These interventions were piloted in a feasibility study [24] prior to this cluster randomised controlled trial (RCT). Learning from this resulted in: (a) more activities being provided for each intervention, (b) distinct age-appropriate resources for primary or secondary school teachers to use and deliver and (c) a greater distinction between ‘mindfulness practices’ and ‘relaxation’.

### Mindfulness Practices

Mental health interventions incorporating mindfulness elements have proven effective in treating and preventing various psychological and physical difficulties [25]. Most research that has been conducted thus far included adult samples; however, there is increasing evidence for the beneficial effects of mindfulness-based interventions (MBI) in youth [26, 27]. More specifically, MBI in youth have been shown to significantly increase positive affect, optimism, attention and social-emotional competence while decreasing dysfunctional behaviour and emotion dysregulation [28]. A number of recent reviews of MBIs in youth have highlighted their impact on cognitive and socio-emotional outcomes [29] including mental health and positive wellbeing, noting that the effects appear to be strongest for emotional problems. Although mindfulness has a rapidly growing evidence base, and a large-scale trial is already taking place in UK secondary schools [30], many of the approaches investigated involve intensive programmes requiring extensive staff training and scheduling in school-based classes. Brief approaches to implementing mindfulness practices could provide a feasible alternative for busy schools.

### Relaxation

Relaxation and mindfulness exercises have long been suggested to incorporate similar underlying processes and thus lead to similar outcomes. However, more recent research has emphasised the significant differences between these two concepts [31]. Relaxation exercises differ from mindfulness exercises in that with the former the individual is asked to focus specifically on relaxation, such as through deep breathing and muscle relaxation, whereas in mindfulness the individual is asked to pay attention to the present moment in a non-judgmental way, such as through meditation [32]. A study conducted by Jain and colleagues [33] relating to a relaxation intervention and a mindfulness intervention found that in adults both interventions led to a significant decrease in distress, while positive mood increased. When applied to young people, there is evidence that both mindfulness and relaxation techniques (RT) can reduce emotional difficulties [34–36].

The effect of RT has been frequently studied in both adults and young people suffering from various acute or chronic medical conditions, such as cancer or asthma [37, 38]. Research investigating the effects of RT with respect to different psychopathological conditions has been lacking. However, there is consistent evidence for the alleviating effects of progressive muscle relaxation on anxiety, stress and depression symptoms in clinical and non-clinical populations [39–41], and for autogenic training on stress and anxiety symptoms [42], and guided imagery on depression, anxiety and stress in psychiatric patients [43].

There are few studies investigating the impact of solely RT on children’s mental health, with some evidence indicating positive effects on anxiety and stress [40]. Relaxation also forms a common thread in many school-based interventions aimed at improving internalising symptoms, including school-based cognitive behavioural therapy (CBT) and Mindfulness programmes [44].

### Strategies for Safety and Wellbeing (SSW)

The development of SSW stemmed from emerging practice in some UK schools around teaching practical approaches to personal safety, known as ‘Protective Behaviours’ (PB). The PB model was developed in the US in 1970 as an anti-victim programme for children, adolescents and adults [45]. The overarching aim of SSW is to increase skills for children around safety, mental health and wellbeing and how to access sources of support. Specifically, pupils are taught to identify (1) what feels safe/unsafe, (2) support networks, (3) coping and help seeking strategies, as well as to (4) recognise and understanding feelings and (5) challenge stigma around mental illness. This is broken down into an 8-week programme. Weeks 1–2 cover the topic ‘It’s safe to talk about mental health’ and weeks 3–5 cover ‘What is safety and knowing when you are not safe?’. Week 6 focusses on ‘Speaking about safety – who could you speak to?’, week 7 focusses on ‘Staying safe in friendships’, while week 8 finishes with ‘Safe ways to manage emotions and network review’. This fits with Personal, Social, Health and Economic Education (PSHE) guidance [46] in the following ways: pupils should be taught to (1) understand how and when they feel unsafe, (2) identify support networks, (3) identify how and from whom to seek help, (4) identify how to recognise and talk about emotions and (5) challenge stereotypes.

Although it has been observed that PB has been applied to schools in the UK [47], there is currently no peer-reviewed evidence for the effectiveness of PB programmes.

## Aims and hypothesis

To date, a mixed picture has emerged, which outlines some potential benefits for Mindfulness Practices and Relaxation, and the need to develop an evidence base for SSW. A scoping exercise, conducted by the Department for Education in England, concluded that all three should be tested to contribute to the UK evidence base for effective interventions to improve mental health in children and young people.

### Effectiveness measurement

#### Primary aims

1. To examine whether Mindfulness Practices are more effective than usual school-based provision in reducing internalising difficulties in young people
2. To examine whether Relaxation is more effective than usual school-based provision in reducing internalising difficulties in young people
3. To examine whether SSW is more effective than usual school-based provision in increasing intended help-seeking behaviour among young people around mental health

#### Primary hypotheses

- H_1_ Young people receiving Mindfulness Practices will report lower internalising difficulties at 3–6 and 9–12 months’ follow-up than those who receive the usual school curriculum
- H_2_ Young people receiving Relaxation will report lower internalising difficulties at 3–6 and 9–12 months’ follow-up than those who receive the usual school curriculum
- H_3_ Young people receiving SSW will report increased intended help-seeking around mental health at 3–6 and 9–12 months’ follow-up than those who receive the usual school curriculum

#### Secondary aims

- To examine the cost-effectiveness of the interventions compared to Usual Practice in terms of the primary outcome measure and paediatric quality of life.

#### Cost effectiveness research questions

1. Are Mindfulness Practices and Relaxation cost-effective when compared to Usual Practice in terms of internalising difficulties and quality of life?
2. Is Strategies for Safety and Wellbeing cost-effective when compared to Usual Practice in terms of intended help seeking and quality of life?

#### Implementation and process evaluation research questions

1. What is the state of participating schools’ existing provision for supporting mental health and wellbeing and their relationship with local mental health services, and does the nature of provision change over the course of the trial?
2. To what extent does implementation follow the guidelines of the specified interventions, e.g., in terms of fidelity and dosage?
3. What is the relationship between implementation variability (e.g., in terms of different levels of fidelity) and intervention outcomes?
4. What are the experiences of schools (pupils and staff) in delivering/receiving Relaxation, Mindfulness Practices and SSW?

## Methods and analysis

The methodology outlined in this protocol follows a similar procedure to that of the AWARE trial [48] in relation to recruitment strategy and the economic evaluation. Both the INSPIRE trial (which this paper describes) and the AWARE trial are being conducted by the same team as part of a wider programme. The Additional file 1 provides an overview of enrollment, intervention and assessment timelines for INSPIRE.

### Design

INSPIRE (INterventions in Schools for Promoting Wellbeing: Research in Education) is a four-arm cluster RCT including three intervention conditions (Mindfulness Practices, Relaxation and SSW) and one wait-list control (Usual Provision).

**Fig. 1:**
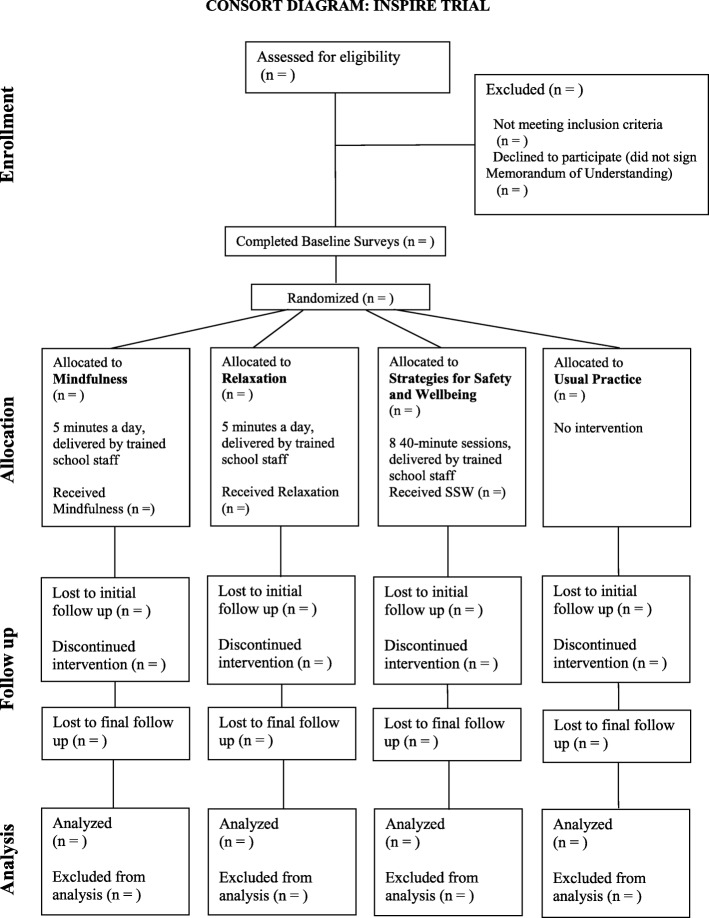
Consolidate Standards of Reporting Trials (CONSORT) diagram

Interventions are delivered to whole school classes as part of the school curriculum. Assessment is undertaken at baseline (prior to intervention randomisation), and then 3–6 months and 9–12 months after interventions have been delivered. Figure 1 outlines a Consolidated Standards of Reporting Trials (CONSORT) Diagram showing the overall trial design.

### Site recruitment

Recruitment of schools began in March 2018 and will close in July 2019. Based on specification from the Department for Education, this study aims to recruit Year-7 and Year-8 pupils (aged 11–13 years) in 64 secondary schools and Year-4 and -5 pupils (aged 8–10 years) in 160 primary schools across England. Within each secondary school, three Year-7 and three Year-8 classes will be required to take part. Primary schools will work with up to four classes (minimum one Year-4 and one Year-5 class).

Schools will be recruited via a range of different networks and mailing lists, including bought data on English schools (school mailings), the Schools in Mind network hosted by the Anna Freud National Centre for Children and Families (AFNCCF), AFNCCF associates and collaborators, the National Institute for Health Research, Public Health England, school commissioners and local authority leads. The project will also be advertised in education publications and on various social media platforms.

Incentives for schools to take part, include:

- £1000 remuneration in recognition of administrative commitments
- The opportunity to introduce whole-class mental health and wellbeing interventions with support from leading experts in child mental health
- The chance to receive free mental health and wellbeing training for selected school staff
- An evaluation feedback report for your school
- Contributing to the wider evidence base on what works for school-based mental health support and how it can best be delivered
- A letter of thanks from the Department for Education acknowledging the school’s important role in this project

### Participant recruitment

Following recruitment of schools, participants in relevant year groups are recruited in two stages. Schools first select delivery groups in each year who will receive an intervention (if allocated). Following this, schools send letters to parents/carers of pupils in these delivery groups. The letter provides information about the study and explains parents/carers’ right to opt their child out of the evaluation. The letter also explains that pupils will only be involved in the study if they assent online before completing the baseline survey. Finally, young people must assent by reading through an online information sheet and ticking boxes agreeing to take part. If they do not assent, they cannot be part of the trial. The first young person joined the trial on the 17 September 2018.

### Inclusion/exclusion criteria

Schools are eligible to participate if:

1. They are a primary school (state-funded/academy/independent) willing to deliver an intervention to one or two Year-4 classes, and one or two Year-5 classes in their school
2. They are a secondary school (state-funded/academy/independent) willing to deliver an intervention to three Year-7 classes and three Year-8 classes in their school
3. They are willing to be allocated to Mindfulness Practices, Relaxation, SSW or continue with usual provision
4. They are willing to allocate 5 min per day for young people to practise these skills for the spring term if allocated to Mindfulness Practices or Relaxation
5. They are willing to allocate eight 40-min lessons to deliver the programme over the spring term if allocated to SSW
6. They are able to send staff to a regional training session, if required
7. They are in England

Young people are eligible to take part if:

8. Their parents/guardians do not withdraw consent
9. They provide assent

Schools are not eligible to take part if:

1. They are a non-mainstream specialist school (e.g., pupil referral unit)
2. They are unable to commit to the study requirements above
3. They are already taking part in similar trials (e.g., MYRIAD [30])
4. They are outside of England

Young people are not eligible to take part if:

1. Their parents do not provide consent for them to take part
2. They do not assent to take part
3. They are not in specified year groups

While privately funded schools are invited to express interest in the project, they will only form < 2% of the total sample. Single-sex schools are also eligible to take part but will be limited to < 5% of the sample.

#### Interventions

Across the active arms of the trial, schools are required to select staff to attend and deliver the interventions. There are no criteria for this role and this can include, but is not limited to: teachers, senior school leaders, teaching assistants, or special educational needs coordinators (SENDCos).

Each of the interventions was developed by a group of experts, consisting of psychologists, researchers, the Programme Director of Mental Health and Wellbeing Schools and a Headteacher Quality Assurance Panel. Teachers who delivered interventions as part of the pilot [24] also provided feedback which was incorporated into interventions delivered in the full trial. Logic models for the interventions are found in the Additional files 2 and 3.

##### Mindfulness Practices

This Mindfulness intervention was developed for the trial by the AFNCCF Schools Programme (lead developer: Dr. Rina Bajaj). It is based on the concept of mindfulness as defined by Kabat-Zinn: ‘paying attention in a particular way: on purpose, in the present moment, and non-judgmentally’ [32], and draws on a number of existing mindfulness models including the RAIN approach [49] and the two-component model of mindfulness [50]. The Mindfulness intervention consists of mindful breathing exercises and other activities focussed on self-awareness of sensations, emotions and thoughts. The exercises are divided into three types: (1) those focussing on the mind; (2) those focussing on the body; (3) those focussing on the world.

School staff complete a half-day face-to-face training course delivered by two AFNCCF professionals that focusses on practising mindfulness exercises. A mindfulness manual – either a primary or secondary school-specific version – is provided. Both manuals contain 21 different activities, as well as suggestions for recommended apps and interactive online games. Mindfulness is delivered to school classes in classrooms for around 5 min each school day at a time chosen by the deliverer, from January to April in the first instance, and this is the period in which implementation is monitored. However, schools are encouraged to continue to practice for 1 year.

##### Relaxation

This Relaxation intervention was also developed for the trial by the AFNCCF Schools Programme (lead developer: Dr. Rina Bajaj). The intervention consists of relaxation exercises focussing on two main themes: (1) deep breathing and (2) progressive muscle relaxation. School staff complete a half-day face-to-face training course focussing on experiential exercises, delivered by AFNCCF professionals. Manuals containing 20 different activities are provided (primary and secondary school versions). These manuals also include recommendations of apps, videos and interactive online games.

Similar to the Mindfulness model, relaxation exercises are delivered in classes for around 5 min each school day, at a time chosen by the deliverer. School staff alternate every week between deep breathing and progressive muscle relaxation activities. Relaxation is delivered to school classes in classrooms from January to April in the first instance, as this is the period in which implementation is monitored. However, schools are encouraged to continue to practise for 1 year.

##### Strategies for Safety and Wellbeing

SSW was also developed by the AFNCCF schools programme (lead developer: Dr. Rina Bajaj), who consulted with experts in PB interventions. School staff complete a half-day face-to-face training course with the lead developer. The training focusses on covering the psychoeducational content of an 8-week session plan with lessons adapted for primary or secondary school pupils. The eight sessions are as follows:

1. It is safe to talk about mental health
2. You are never too young to talk mental health (primary schools)/We all have mental health (secondary schools)
3. What is safety?
4. Early warning signs – noticing our bodies
5. Early warning signs – noticing our feelings and thoughts
6. Developing our safety networks
7. Safe friendships
8. Safe ways of managing emotions

Each session lasts for approximately 40 min and is delivered once a week for 8 weeks.

##### Usual Practice

Schools allocated to the Usual Practice group are not required to deliver a specific mental health intervention during the programme (June 2018 to January 2021), but may already do so as part of their usual whole-school provision around mental health. All participating schools will complete the Usual Provision Survey at the end of the project (second follow-up) so we are able to track changes in mental health and wellbeing provision. At the end of the project, schools in the Usual Practice arm will select from a suite of training available at the AFNCCF and send up to six staff members on their chosen training.

### Study measures

The following measures will be completed prior to the intervention and follow-up will take place at 3–6 and 9–12 months post intervention. All questionnaires will be completed online.

### Pupils

#### Primary outcome measures

- For Mindfulness Practices and Relaxation, the primary outcome measure is internalising difficulties as measured by the Short Mood and Feelings Questionnaire (SMFQ) [51]
- For SSW the primary outcome measure is intended help-seeking, as measured by the General Help-Seeking Questionnaire (GHSQ) [52]

#### Secondary outcome measures

The secondary outcome measures across all interventions include:

- Mental health first aid [53]
- Paediatric Quality of Life (Child Health Utility-9D; CHU9D) [54]
- Positive wellbeing: Huebner Life Satisfaction Scale (LSS) [55]

In addition, secondary school pupils will be asked further questions:

- Stigma (knowledge): Mental Health Knowledge Schedule (MAKS) [56]
- Stigma (behaviour): Reported and Intended Behaviour Scale (RIBS) [57]
- Stigma (attitudes): Attitudes towards mental health [58]
- Behavioural problems: Me & My Feelings questionnaire [59]^1^
- Support from school staff: Student Resilience Survey (SRS) School Connection subscale [60]^1^

### School staff

Similar to pupils, school staff participating in the project (those who are nominated by the school to deliver the intervention) will complete measures around mental health literacy [61–65] prior to the intervention. Follow-up will take place at 3–6 and 9–12 months post intervention and all questionnaires will be completed online.

### Measures for economic evaluation

As part of the assessment, pupils will complete:

- A Client Service Receipt of Inventory (CSRI; adapted for the study population) [66]
- A Service Information Schedule (SIS) [67]

In addition to this, school staff delivering the interventions and school finance officers will provide the following data informing the calculation of an intervention cost: time spent preparing and delivering the intervention, staff member salary band, staff member full-time equivalent working hours, staff member pension contributions and national insurance contributions as a percent of their annual salary, and any other staff overheads.

### Implementation and process monitoring measures

#### Usual Provision Survey

Before intervention delivery, and again 1 year later, a senior leader in each school will be asked to complete a survey online regarding current whole-school mental health provision.

#### Implementation surveys and outcome measures

School staff that deliver an intervention will complete one online implementation survey per delivery group at the end of the initial delivery period. Questions will cover six key aspects of implementation, namely fidelity, quality, dosage, participant responsiveness, reach and adaptations. Within this, three aspects relating to the social validity of the intervention (acceptability, feasibility and utility) will also be assessed using a standardised questionnaire [68]. The survey will also capture other aspects related to dosage and the time of day that the intervention was delivered.

#### Qualitative data and observations

Qualitative implementation and process data will be collected at two time points. The first time point will take place at mid- to late-implementation of each of the interventions. Twelve schools will be recruited from the main sample as qualitative case study schools; one school per intervention in each of the four areas of England (north west, north east, south west, south east). This will not include Usual Practice schools. Case study schools will be recruited via expression of interest, to maximise the likelihood of engagement with the qualitative research, and sampled based on variation in their usual provision around mental health, drawing on data from two items in the first Usual Provision Survey:

1. Please identify, in the last 2 years, the activities and approaches that have been used in your school and indicate who has delivered/provided these activities
2. How significant are the following potential barriers to providing effective
  mental health support within your school?

While the case study schools could be selected on multiple bases, these contextual factors are those of particular interest to the trial, in terms of how they could affect the implementation and take-up of the interventions within the schools.

Face-to-face or telephone interviews will be conducted with two to three members of staff (including a school senior leadership team member and a staff member delivering the intervention) and one to two focus groups will be conducted face-to-face with pupils (with approximately four to five pupils in each focus group) at each school. Pupils will be selected via expression of interest, and up to 10 will be invited to participate due to risk of attrition or pupils declining to take part. Learning from the feasibility study [24] indicated that this sample size would yield a large amount of rich qualitative data, while still being manageable in terms of the research team’s capacity.

The interviews/focus groups will be semi-structured, enabling the research team to guide the conversation according to their topics of interest, while at the same time allowing participants to raise issues around these topics that are pertinent to them. All interviews/focus groups will be audio-recorded and transcribed verbatim.

The topics that the interviews with staff will cover include:

1. Experiences of delivering the interventions and receiving training to deliver the interventions
2. Perceptions of the barriers and facilitators to delivery
3. Perceptions of impact
4. Suggestions for improvement of the interventions
5. Barriers and facilitators to the sustainability of the interventions

The topics that the focus groups with pupils will cover include:

1. Experiences of taking part in the interventions
2. Perceptions of impact and helpful aspects of the interventions
3. Suggestions for improvement of the interventions

A session of the intervention at each school will also be observed by the research team to gather contextual information about what the interventions look like on the ground. Field notes will be taken during the observation on the process of delivery, the layout of the room, and the atmosphere during delivery. Individual pupil or staff responses will not be recorded.

The second time point will take place approximately 9–12 months after Time 1. At Time 2, we will conduct approximately five follow-up visits with five of the schools from Time 1 at which, according to implementation monitoring survey data, the interventions have been particularly well embedded to explore long-term impact and facilitators to (ongoing) implementation from staff and pupil perspectives. This will involve face-to-face or telephone interviews with one to two members of staff and one face-to-face focus group with pupils at each school. We will also explore potential barriers to long-term impact and implementation through a telephone interview with a staff member at approximately three schools at which, again according to implementation monitoring survey data, the interventions have not been particularly well embedded.

Furthermore, as schools that express interest in taking part as a case study are likely to be the more engaged schools, at the second time point we will also conduct a small number of telephone interviews with staff at schools that have engaged less with the trial in general. This will allow us to gather data on the barriers that they may have experienced to engaging with the trial and could include schools that have dropped out of the trial.

### Randomisation of schools

To ensure approximate distribution across conditions, randomisation will be carried out by Kings Clinical Trials Unit (KCTU). Due to recruitment rates the trial is split into two cohorts. Randomisation of schools will take place in two batches (first cohort: 22 and 23 October 2018; second cohort planned for 21 and 22 October 2019). In both randomisations minimisation will be used to take into account regional representation (four recruitment hubs); deprivation as indicated by free school-meal (FSM) eligibility (tertiles of sample FSM rates); current mental health provision (Mindfulness, Relaxation, Strategies for Safety and Wellbeing, other structured lessons; none); and urban/rural situation of school. Only the statistician, quantitative data analyst and economist are blind to intervention allocation.

### Data management

All quantitative data will be stored on the University of Manchester’s secure server. The Data Manager (JS), along with the Research Assistants (EA and RM) will be responsible for cleaning and coding the data. Qualitative data (audio files and transcripts) will be stored on the AFNCCF’s secure server. The Qualitative Research Lead (ES), supported by the Trials Manager (DH), Research Officer (AM) and Research Assistants (RM and EA), will be responsible for data storage, and checking transcripts and ensuring their accuracy.

### Sample size

The trial will be analysed on class-level, controlling for school- and class-level clustering, due to the delivery of the intervention within classes. The design for the current trial is a between-school trial. To increase the efficiency of the design, we will use a single set of control schools as a comparator for all three interventions. The schools will be randomised to four groups (Usual Practice, Mindfulness Practices, SSW, Relaxation); around two to three of the schools in each arm will be primary schools and one to three will be secondary schools to accommodate the different numbers of classes and class sizes within each school type.

While cluster effects of emotional distress on school level are usually small [69, 70], no data on class-level clustering were available. To our knowledge, so far no study has looked into school-level intra-class correlations (ICCs) of help-seeking. We conducted a pilot study with *N* = 2289 students nested within 113 classes within 17 schools and we found ICCs of .05 for the SMFQ and of .03 for the GHSQ (with upper borders of bootstrapped 95% confidence intervals of .12 for the GHSQ and .13 for the SMFQ). The following sample size calculation is based on an ICC of *ρ* = .15, which is still conservative given the estimates found in the pilot (for a significance level of *p* = .05 and statistical power of *β* = .80).

Pre-test values of the outcome measures will be used as predictors of within-school variance. Since pre- and post-tests tend to be correlated, a conservative estimate of *R*^2^ = .20 was used. Since only a small effect due to the intervention is expected, *MDES* = .20 was selected as the target effect size. On average, we assume primary schools to have two classes (with *N* = 25 students each) and secondary schools to have six classes (*N* = 20 students each). Finally, since the analysis of the primary outcome only compares each active treatment individually against the control arm, no correction of error rates was performed for these pre-planned directed hypotheses [71].

Accommodating the setting of a delivery from four different study areas, we aim to recruit 56 schools per arm (40 primary; 16 secondary with six classes each). Given this number of classes, the MDES without controlling for any additional variables in the full sample is *MDES* = .129 (*MDES* = .190 in primary and MDES = .177 in secondary schools only). Including pre-tests leads to an MDES = .127 for the full sample (and MDES = .186 in primary and MDES = .173 in secondary schools). The statistical analyses will be undertaken by an independent statistician (JB) at the University of Dundee.

### Statistical analysis of the primary outcome

A detailed statistical analysis plan will be written prospectively, but the sample size calculation was based on estimating three mixed models, each comparing an active treatment with the control arm. The mixed model will allow for school-level clustering; control for baseline levels in the primary outcome; and the minimisation variables (see above). An intervention will be evaluated as potentially effective if the point estimate of the coefficient for the dummy variable coding the difference between intervention and control arm indicates a group difference in the hypothesised direction for the outcome and the cluster-bootstrapped 95% confidence interval does not include zero. The primary outcome tested for the Mindfulness Practices and Relaxation arms is the SMFQ; and for SSW the primary outcome is the GHSQ; all three at 3–6 months’ follow-up. The analysis will be intent-to-treat. This analysis will be undertaken using R [72]. The potential impact of missing data will be evaluated in a sensitivity analysis using fully conditional specification [73].

### Economic evaluation

#### Service use and costs

A Service Information Schedule will be designed to facilitate micro-costing of the interventions. Information on services and supports used by the young people in the study will be collected using a specially adapted version of the CSRI [66]. From these data, we will investigate whether patterns of service use and associated costs differ, and explore whether any differences are driven by individual characteristics or baseline level of need.

#### Cost-effectiveness analysis

To assess whether the interventions are cost-effective relative to Usual Practice, cost-effectiveness and cost-utility analyses will be undertaken for change in (a) the primary outcome measure for each intervention and (b) quality-adjusted life years (derived from the CHU9D) [54]. We will employ an analytical approach that allows for adjustment for confounders, the likely non-normal distribution of cost data, the joint analysis of cost and outcome measures and the potential effects of clustering. Results will be presented as cost-effectiveness acceptability curves [74] plotting the probability that the intervention will be considered cost-effective compared to treatment as usual against different levels of willingness to pay for an improvement in outcome. Sensitivity analyses will be undertaken by varying assumptions used to calculate the intervention cost. Potential sub-group analyses will be identified post hoc.

### Process and implementation analysis

Descriptive statistics will be used to document usual school provision and how this changes over the course of the project, as well as to document the implementation of Relaxation, Mindfulness Practices and SSW. Additionally, for examining implementation, we will compare ‘intervention as delivered’ from our survey data with ‘intervention as planned’. Where applicable, the latter can be used to determine the proportion of participating schools that can be deemed to have achieved at least a minimum standard of intervention delivery (e.g., ‘on treatment’ status). To assess the relationship between implementation variability and outcomes, multi-level modelling will be used, in which we fit the implementation data noted above (or on treatment status derived from said data) as explanatory variables at the school or class level, to assess the extent to which they are predictive of intervention outcomes at the pupil level.

Qualitative interviews and focus group transcripts will be analysed using thematic analysis [75], using the NVivo version 12 [76] data analysis software package. Up to three members of the research team will initially code or assign relevant extracts of the transcripts to broad overarching categories, derived ‘top-down’ from the research questions (e.g., suggestions for improvement). The researchers will then break down the data (transcript extracts) coded within these overarching categories into themes and subthemes, derived ‘bottom-up’ from the data. A fourth member of the research team will then re-code 10–20% of the transcripts using the themes and subthemes derived from the data by the other members of the team. The purpose of the latter step is to help the original researchers to refine and reflect on their themes and subthemes, with the additional researcher suggesting edits or additions where necessary.

### Patient and public involvement

Young people provided input into the development and refinements of the interventions, including what techniques and activities should be included, as well as input into the design of the booklets. In relation to research, young people provided input into what questions were included in the final questionnaires for young people and will be involved in disseminating reports and findings.

## Trial status

Recruitment for schools opened in March 2018 and will stay open until July 2019. The first young person joined the trial on 17 September 2018. The last participants will be followed up at the 1-year follow-up in January/February 2021. A timeline for the trial is available as Additional file 4.

### Protocol

V1, 14 January 2019. Substantial changes to the protocol will be communicated from the Trials Manager to relevant parties (e.g., ISRCTN). The protocol follows Standard Protocol items: Recommendations for Interventional Trials (SPIRIT) reporting [77].

## Supplementary information

**Additional file 1.** INSPIRE Trial Timeline.

**Additional file 2.** Logic model for Mindfulness and Relaxation.

**Additional file 3.** Logic model for Strategies for Safety and Wellbeing.

**Additional file 4.** Reporting checklist for protocol of a clinical trial.

## Data Availability

An anonymised quantitative dataset generated and/or analysed during the current study will be available in 2022 once the study has finished. A decision regarding storage location is yet to be finalised, please contact the Principal Investigator or Trials Manager for further information.

## Footnotes

1 These measures were included in baseline primary surveys as they are thought to be potential moderators. However, these are removed at follow-up due to data burden for primary schools.
