# Supplementary material for: Promoting mental health and wellbeing in schools: examining Mindfulness, Relaxation and Strategies for Safety and Wellbeing in English primary and secondary schools: study protocol for a multi-school, cluster randomised controlled trial (INSPIRE)
Source: Trials. 2019 Nov 21;20:640. doi: 10.1186/s13063-019-3762-0 (PMC6868714; doi:10.1186/s13063-019-3762-0)
Supplement: Supplementary file 2 — Additional file 2. Logic model for Mindfulness and Relaxation. [file 13063_2019_3762_MOESM2_ESM.pptx]

## Slide 1
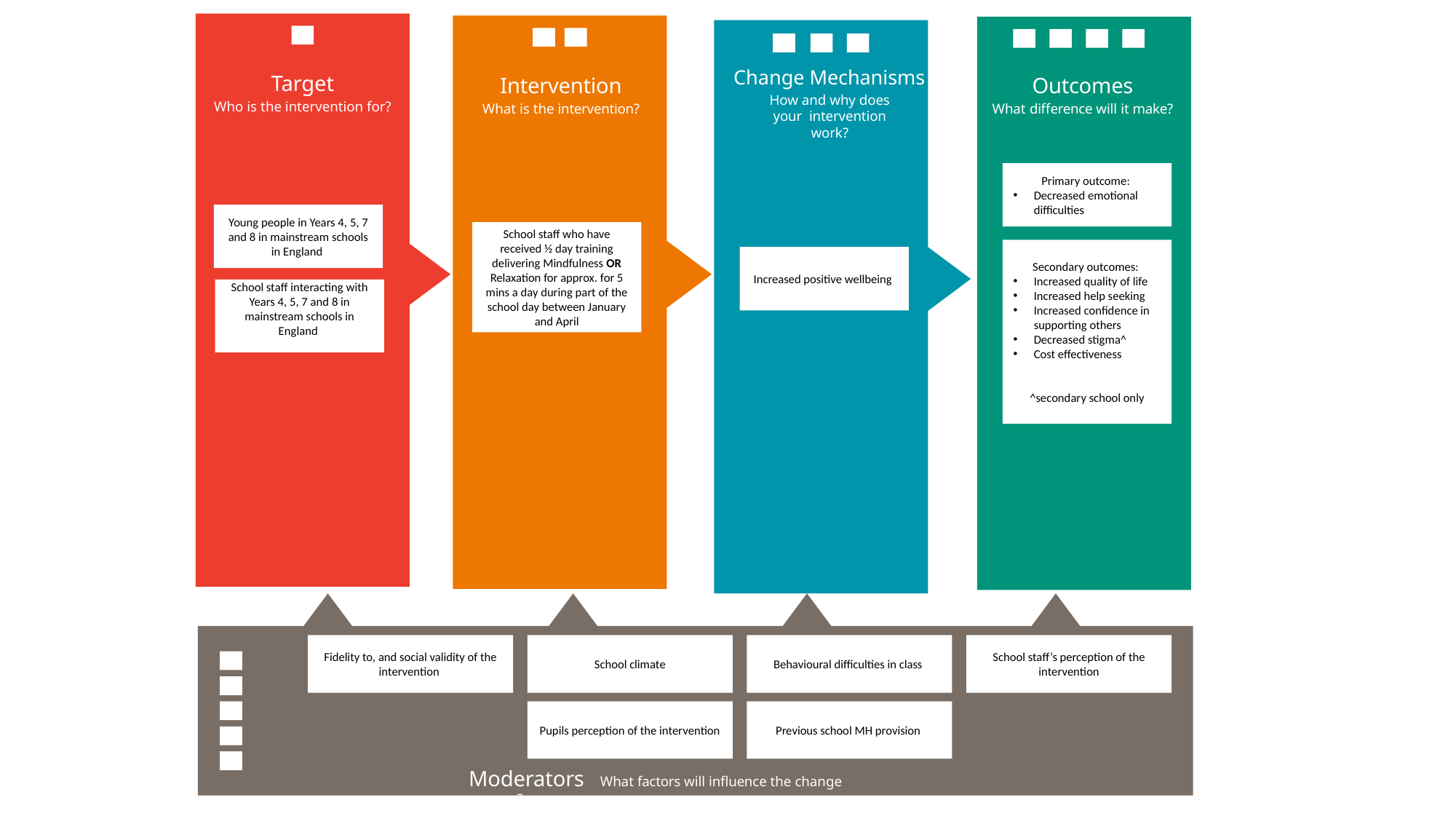

Target
Who is the intervention for?
Intervention
What is the intervention?
Outcomes
What difference will it make?
Change Mechanisms
How and why does your intervention work?
Primary outcome:
Decreased emotional difficulties
Young people in Years 4, 5, 7 and 8 in mainstream schools in England
School staff who have received ½ day training delivering Mindfulness OR Relaxation for approx. for 5 mins a day during part of the school day between January and April
Secondary outcomes:
Increased quality of life
Increased help seeking
Increased confidence in supporting others
Decreased stigma^
Cost effectiveness
^secondary school only
Increased positive wellbeing
School staff interacting with Years 4, 5, 7 and 8 in mainstream schools in England
Fidelity to, and social validity of the intervention
School climate
Behavioural difficulties in class
School staff’s perception of the intervention
Pupils perception of the intervention
Previous school MH provision
Moderators What factors will influence the change process?
