# Supplementary material for: Promoting mental health and wellbeing in schools: examining Mindfulness, Relaxation and Strategies for Safety and Wellbeing in English primary and secondary schools: study protocol for a multi-school, cluster randomised controlled trial (INSPIRE)
Source: Trials. 2019 Nov 21;20:640. doi: 10.1186/s13063-019-3762-0 (PMC6868714; doi:10.1186/s13063-019-3762-0)
Supplement: Supplementary file 3 — Additional file 3. Logic model for Strategies for Safety and Wellbeing. [file 13063_2019_3762_MOESM3_ESM.pptx]

## Slide 1
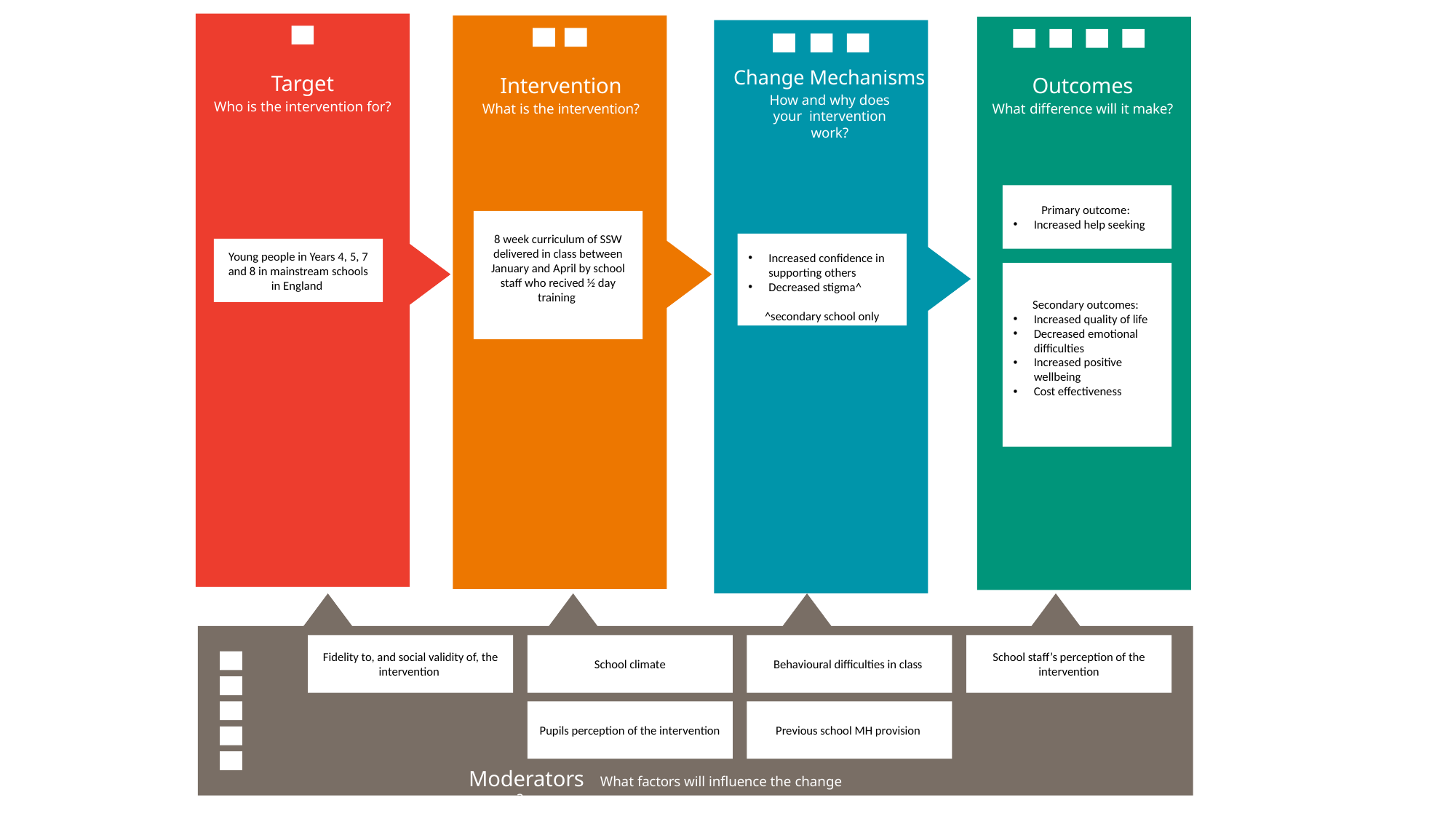

Target
Who is the intervention for?
Intervention
What is the intervention?
Outcomes
What difference will it make?
Change Mechanisms
How and why does your intervention work?
Primary outcome:
Increased help seeking
8 week curriculum of SSW delivered in class between January and April by school staff who recived ½ day training
Increased confidence in supporting others
Decreased stigma^
^secondary school only
Young people in Years 4, 5, 7 and 8 in mainstream schools in England
Secondary outcomes:
Increased quality of life
Decreased emotional difficulties
Increased positive wellbeing
Cost effectiveness
Fidelity to, and social validity of, the intervention
School climate
Behavioural difficulties in class
School staff’s perception of the intervention
Pupils perception of the intervention
Previous school MH provision
Moderators What factors will influence the change process?
