# Supplementary material for: Promoting mental health and wellbeing in schools: examining Mindfulness, Relaxation and Strategies for Safety and Wellbeing in English primary and secondary schools: study protocol for a multi-school, cluster randomised controlled trial (INSPIRE)
Source: Trials. 2019 Nov 21;20:640. doi: 10.1186/s13063-019-3762-0 (PMC6868714; doi:10.1186/s13063-019-3762-0)
Supplement: Supplementary file 4 — Additional file 4. Reporting checklist for protocol of a clinical trial. [file 13063_2019_3762_MOESM4_ESM.doc]

Schedule of enrolment, intervention, and assessments for AWARE. P = Pupil, SM = Staff Member, PG = Parent/Guardian

|  |  | |  |  | | | **STUDY PERIOD** | | | | | | | | | | | | | | | | | | | | | |
| --- | --- | --- | --- | --- | --- | --- | --- | --- | --- | --- | --- | --- | --- | --- | --- | --- | --- | --- | --- | --- | --- | --- | --- | --- | --- | --- | --- | --- |
|  | **Enrolment**  **(months)** | | **Allocation**  **(months)** |  | | | **(months)** | | | | | | | | | | | | | | | | | | | | | |
| **TIMEPOINT**** | ***-7 to***  ***-2*** | ***-1*** | ***0*** | ***1*** | ***2*** | | | ***3*** | | ***4*** | | ***5*** | | ***6*** | | ***7*** | | ***8*** | | ***9*** | | ***10*** | ***11*** | ***12*** | ***13*** | ***14*** | ***15*** | ***16*** |
| **ENROLMENT:** |  |  |  |  |  | | |  | |  | |  | |  | |  | |  | |  | |  |  |  |  |  |  |  |
| **School eligibility screen (EoI)** | X |  |  |  |  | | |  | |  | |  | |  | |  | |  | |  | |  |  |  |  |  |  |  |
| **School completes MOU and DSA** | X |  |  |  |  | | |  | |  | |  | |  | |  | |  | |  | |  |  |  |  |  |  |  |
| **Consent/Assent** | PG | CYP |  |  |  | | |  | |  | |  | |  | |  | |  | |  | |  |  |  |  |  |  |  |
| **Allocation** |  |  | X |  |  | | |  | |  | |  | |  | |  | |  | |  | |  |  |  |  |  |  |  |
| **INTERVENTIONS** |  |  |  |  |  | | |  | |  | |  | |  | |  | |  | |  | |  |  |  |  |  |  |  |
| ***School staff training in interventions*** |  |  |  |  |  | | |  | |  | |  | |  | |  | |  | |  | |  |  |  |  |  |  |  |
| ***Mindfulness*** |  |  |  |  |  | | |  | |  | |  | |  | |  | |  | |  | |  |  |  |  |  |  |  |
| ***Relaxation*** |  |  |  |  |  | | |  | |  | |  | |  | |  | |  | |  | |  |  |  |  |  |  |  |
| ***Strategies for Safety and Wellbeing*** |  |  |  |  |  | | |  | |  | |  | |  | |  | |  | |  | |  |  |  |  |  |  |  |
| ***Usual Provision*** |  |  |  |  |  | | |  | |  | |  | |  | |  | |  | |  | |  |  |  |  |  |  |  |
| **TIMEPOINT**** | ***-7 to***  ***-2*** | ***-1*** | ***0*** | ***1*** | | ***2*** | | | ***3*** | | ***4*** | | ***5*** | | ***6*** | | ***7*** | | ***8*** | | ***9*** | ***10*** | ***11*** | ***12*** | ***13*** | ***14*** | ***15*** | ***16*** |
| **ASSESSMENTS:** |  |  |  |  | |  | | |  | |  | |  | |  | |  | |  | |  |  |  |  |  |  |  |  |
| ***Usual Provision survey***^b^ | X | X |  |  | |  | | |  | |  | |  | |  | |  | |  | |  |  |  |  |  |  |  |  |
| ***SMFQ^a^*** |  | X |  |  | |  | | |  | |  | |  | |  | |  | |  | |  |  |  |  |  |  |  |  |
| ***GHSQ^a^*** |  | X |  |  | |  | | |  | |  | |  | |  | |  | |  | |  |  |  |  |  |  |  |  |
| ***MHFA^a^*** |  | X |  |  | |  | | |  | |  | |  | |  | |  | |  | |  |  |  |  |  |  |  |  |
| ***CHU9D^a^*** |  | X |  |  | |  | | |  | |  | |  | |  | |  | |  | |  |  |  |  |  |  |  |  |
| ***LSS^a^*** |  | X |  |  | |  | | |  | |  | |  | |  | |  | |  | |  |  |  |  |  |  |  |  |
| ***MAKS^a*^*** |  | X |  |  | |  | | |  | |  | |  | |  | |  | |  | |  |  |  |  |  |  |  |  |
| ***RIBS^a*^*** |  | X |  |  | |  | | |  | |  | |  | |  | |  | |  | |  |  |  |  |  |  |  |  |
| ***Attitudes^a*^*** |  | X |  |  | |  | | |  | |  | |  | |  | |  | |  | |  |  |  |  |  |  |  |  |
| ***M&MFa^*** |  | X |  |  | |  | | |  | |  | |  | |  | |  | |  | |  |  |  |  |  |  |  |  |
| ***SRSa^*** |  | X |  |  | |  | | |  | |  | |  | |  | |  | |  | |  |  |  |  |  |  |  |  |
| ***CSRI^a^*** |  | X |  |  | |  | | |  | |  | |  | |  | |  | |  | |  |  |  |  |  |  |  |  |
| ***SIS^b^*** |  |  |  |  | |  | | |  | |  | |  | |  | |  | |  | |  |  |  |  |  |  |  |  |
| ***Mental health literacy^b^*** |  | X |  |  | |  | | |  | |  | |  | |  | |  | |  | |  |  |  |  | X |  |  |  |
| **TIMEPOINT**** | ***-7 to***  ***-2*** | ***-1*** | ***0*** | ***1*** | | ***2*** | | | ***3*** | | ***4*** | | ***5*** | | ***6*** | | ***7*** | | ***8*** | | ***9*** | ***10*** | ***11*** | ***12*** | ***13*** | ***14*** | ***15*** | ***16*** |
| ***Implementation survey ^b^*** |  |  |  |  | |  | | |  | |  | |  | |  | |  | |  | |  |  |  |  |  |  |  |  |
| ***Sustainability survey ^b^*** |  |  |  |  | |  | | |  | |  | |  | |  | |  | |  | |  |  |  |  | X |  |  |  |
| ***Qualitative interviews*** |  |  |  |  | |  | | |  | |  | |  | |  | |  | |  | |  |  |  |  |  |  |  |  |
| ***Observations*** |  |  |  |  | |  | | |  | |  | |  | |  | |  | |  | |  |  |  |  |  |  |  |  |

^a^ Young person completes

^b^ School staff member completes

*Secondary school only

^baseline only for primary schools
